# Epidemiology of urinary tract infection and antimicrobial resistance in a pediatric hospital in Nepal

**DOI:** 10.1186/s12879-019-3997-0

**Published:** 2019-05-14

**Authors:** Rabina Ganesh, Dhiraj Shrestha, Balkrishna Bhattachan, Ganesh Rai

**Affiliations:** 1 Department of Microbiology, Siddhi Memorial Hospital, Bhaktapur, Nepal; 2 Department of Microbiology, Shi-Gan International College of Science and Technology, Kathmandu, Nepal; 3 Department of Microbiology, Tri-Chandra Multiple College, Kathmandu, Nepal

**Keywords:** Antimicrobial resistance, *E. coli*, *Klebsiella* spp., Nepal, Urinary tract infection

## Abstract

**Background:**

Urinary tract infection is an infection affecting infants and children. The aim of this study was to determine the etiology of urinary tract infection along with their antimicrobial resistance.

**Methods:**

This cross-sectional study was conducted from June 2015 to January 2016 at Siddhi Memorial Hospital, Bhaktapur, Nepal. Urine samples were first cultured on cystine lactose electrolyte deficient agar and blood agar by semi-quantitative technique, and then incubated aerobically for 18–24 h at 37 °C. The identified bacterial isolates were tested for antimicrobial susceptibility by Kirby Bauer disc diffusion technique.

**Results:**

Of 1599 urine samples, 12.3% samples showed significant bacterial growth. *E. coli* (58.7%) was the most common pathogen, followed by *Klebsiella pneumoniae* (22.5%). Most of the isolates were resistant to ampicillin and co-trimoxazole, while least were resistant to amikacin and nitrofurantoin. Higher multi-drug resistance (61.9%) was observed among isolates.

**Conclusions:**

*E. coli* and *Klebsiella* spp. were predominant cause of pediatric urinary tract infection in children. Higher susceptibility observed against aminoglycosides and nitrofurans make these drugs suitable in emergency.

## Background

Urinary tract infection (UTI) is any infection leading to an inflammatory response in the epithelium of the urinary tract [1]. UTIs affect both males and females of all ages. The occurrences of UTIs are higher in women, which are likely caused by anatomical differences, hormonal effects and behaviors [2]. The cases of UTI among Nepalese patients attending general hospitals ranges from 23.1 to 37.4% [3]. Bacteria are the common etiology of UTIs accounting more than 95% of the cases. *Escherichia coli* is the most common causative organisms of UTI and is solely responsible for more than 80% of UTI [4].

This study was conducted among children with symptoms of UTI visiting a tertiary healthcare in Nepal to determine spectra of uropathogens, especially *E. coli* and *Klebsiella* spp., and their antimicrobial resistance (AMR). This would help the clinician in using appropriate antibiotics for the clinical management of UTI.

## Methods

### Study design, area and sample population

The cross-sectional study was conducted from June 2015 to January 2016 in Siddhi Memorial Hospital, Bhaktapur, Nepal. The Hospital is the only children hospital in the district and serves infants and children patients of the district and other nearby areas. The totals of 1599 urine samples were included in this study. The study populations were infants and children patients not exceeding 14 years age, seeking treatment at the hospital with symptoms of UTI. The symptoms included fever along with dysuria and/or loss of bladder control and/or lower back pain and/or cloudy or foul smelling urine. For infants and younger children symptoms included was fever and parental reporting of cloudy or foul smelling urine. The clean catch urine samples were collected in sterile container. In infants and non-toilet-trained children, a sterile foil bowl was placed underneath the genitalia for clean-catch urine collection. If this was not possible, a plastic bag was attached to genitalia for urine collection. In toilet-trained children, a clean-catch voided midstream urine sample was collected. In both cases the genitalia was cleaned beforehand to reduce contamination. Invasive techniques were avoided for urine collection. Children receiving antimicrobial drugs treatment and children undergoing bladder catheterization within 48 h were excluded in the study. The study was ethically approved by the Institutional Review Committee of Shi-Gan International College of Science and Technology (SICOST).

### Laboratory examinations of samples

#### Macroscopic examination of urine

Urine color was observed macroscopically immediately after collection for possible signs of contaminations.

#### Processing of samples and identification of the isolates

First of all, 1 μL of all urine samples were streaked on cystine lactose electrolyte deficient (CLED) agar (HiMedia Pvt. Ltd., India) and blood agar (HiMedia Pvt. Ltd., India) by semi-quantitative method using calibrated loop. The plates were then incubated aerobically for 18–48 h at 37 °C and growths were observed. Positive culture result was considered for plates showing 100 or more colonies i.e. more than or equal to 10^5^ colony forming units (CFU)/ml [5]. Identifications of isolates were done considering Gram’s staining morphology, cultural characteristics and biochemical properties [6].

#### Antimicrobial susceptibility testing

All identified isolates of *E. coli* and *Klebsiella* spp. were tested for susceptibility against amikacin (30 μg), ampicillin (10 μg), cephalexin (30 μg), cefixime (10 μg), cefpodoxime (30 μg), co-trimoxazole (1.25/23.75 μg), nitrofurantoin (300 μg), ofloxacin (5 μg) (HiMedia Pvt. Ltd., India) using Kirby Bauer disc diffusion technique on Mueller-Hinton agar (HiMedia Pvt. Ltd., India). The results were interpreted using CLSI 2015 [7]. Quality control was accessed using *E. coli* ATCC 25922. Isolates resisting two or more classes of antimicrobial agents were considered multidrug resistant (MDR) [8].

#### Data management and statistical analysis

Data obtained were entered and managed in Microsoft Excel (version 2010, Microsoft Corporation, USA), and relation of variables were analyzed using ratio and percentage.

## Results

Among total 1599 urine samples processed, 929 (58.1%) samples were collected from male children while 668 (41.8%) samples from female children. Of 1599 samples, only 197 (12.3%) samples were culture positive and hence UTI. Of 197 UTI cases, UTIs were higher in female children, i.e. 120 (60.9%) compared to male children, i.e. 77 (39.1%). UTIs were highest in age group 2–5 years (Table 1).

**Table 1:** Gender and age distribution of UTIs among children

Age group (years)	Male (%)	Female (%)	Total
≤1	24 (43.6)	31 (56.3)	55
2–5	33 (36.6)	57 (63.3)	90
6–9	17 (41.4)	24 (58.5)	41
10–14	3 (27.2)	8 (72.7)	11
Total	**77 (39.1)**	**120 (60.9)**	**197**

Rate of UTIs in inpatients and outpatients were similar (Table 2).

**Table 2:** Distribution of UTIs in children

Types of patient	Total patients	UTI positive	%
Inpatient	159	20	12.6
Outpatient	1440	177	12.3
Total	1599	197	12.3

**Fig. 1:**
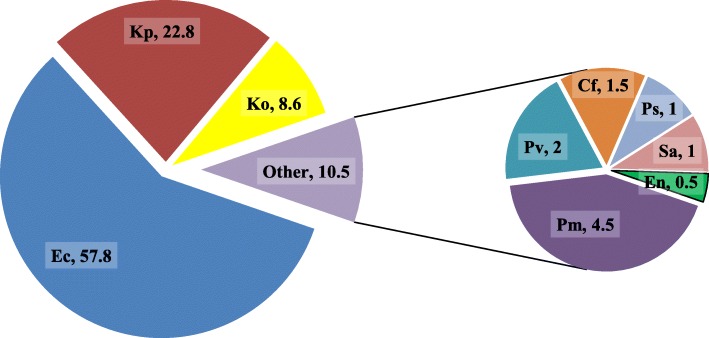
Identified bacterial isolates in UTI patients (%). Legends (as represented by different colors in pie diagram): Each color represent the fraction of the total isolated bacteria in percentage namely, Ec = *E. coli,* Kp = *K. pneumoniae,* Ko = *K. oxytoca,* Pm = *P. mirabilis,* Pv = *P. vulgaris,* Cf = *C. freundii,* Ps = Pseudomonas spp., Sa = *S. aureus,* En = *Enterococcus* spp.

Total of seven different species of Gram negative bacteria were isolated. Among them, *E. coli,* 114 (57.8%) was predominant followed by *Klebsiella* spp., 62 (31.4%) (Fig. 1).

**Fig. 2:**
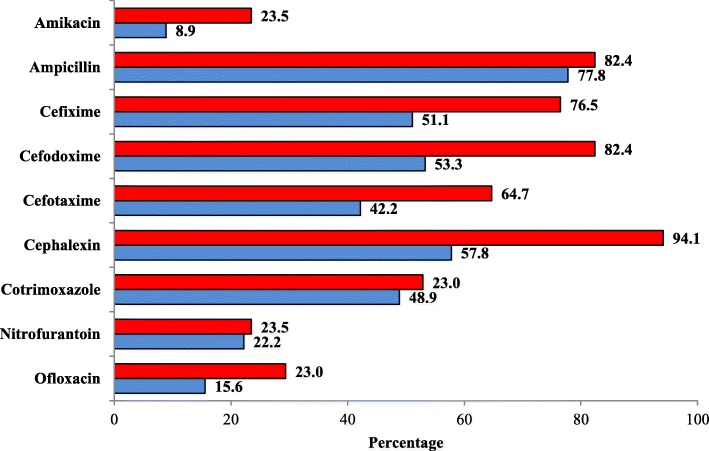
Antibiotic resistivity of *K. pneumoniae* and *K. oxytoca.* Legends (as represented by two colors): Red color represents the resistivity percentage of *K. oxytoca* against the antibiotics. Blue color represents the resistivity percentage of *K. pneumoniae* against the antibiotics

**Fig. 3:**
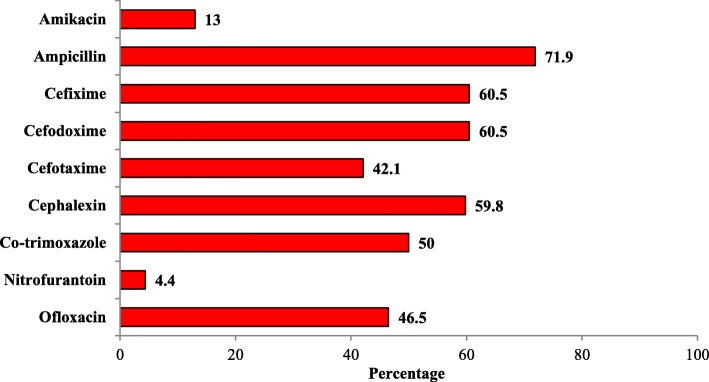
Antibiotic resistivity of *E. coli.* Legends (as represented by red color): Red color represents the resistivity percentage of *E. coli* against the antibiotics

Most *K. oxytoca* isolates were resistant to cephalexin, 16 (94.1%) and least were resistant to nitrofurantoin, 4 (23.5%) and amikacin, 4 (23.5%). Likewise, most *K. pneumoniae* isolates were resistant to ampicillin, 35 (77.8%) and least were resistant to amikacin, 4 (8.9) (Fig. 2). Most *E. coli* isolates were resistant to ampicillin, 82 (71.9%) and least were resistant to nitrofurantoin, 5 (4.4%) (Fig. 3). Higher score of MDR, 109 (61.9%), was observed among isolates (Table 3).

**Table 3:** MDR among isolates

Bacteria	Total isolates	MDR isolates (%)
*E. coli*	114	72 (63.2)
*Klebsiella pneumoniae*	45	26 (57.8)
*K. oxytoca*	17	11 (64.7)
Total	176	109 (61.9)

## Discussion

UTIs are frequent in children and may incur adverse consequences [9]. Thus, reliable diagnosis of UTI in children is critical. In our study, only 12.3% of the 1599 urine samples yielded culture positive result. This warrants discordance of clinical and laboratory examination in the hospital. Laboratories choose one diagnostic threshold but there is no universally accepted threshold. We used the threshold of 10^5^ CFU/ml to determine culture positive result. This can miss lower bacterial counts. Higher threshold could have decreased the culture positive result. Lowering the threshold to 10^4^ CFU/ml can produce clinically valuable results [10].

The study population had higher male children compared to female children (male to female ratio 3:2). This was the involuntary recruitment bias. The other bias could be greater parental urge and/or greater doctors’ preference in testing male children than female children. Also, this could be due to greater difficulty in clinically diagnosing male UTI. On contrast, UTIs were higher in female children as compared to male children (female to male ratio = 3:2). UTIs are more common in girls compared to boys due to shorter length of urethra [11]. Also, this could be due to better clinical diagnosis of UTI in girls than boys by doctors. Or this could be due to more likelihood of growth from specimens collected from females, perhaps due to higher rates of contamination of male specimens. This concords with other reports [3, 4, 9, 12]. With the increase in age, female children showed considerable increase in UTI compared to male children. Majority of UTIs were seen in the ages below 6 years of age. Likewise, rate of UTIs was fairly similar in outpatients and inpatients.

Among seven identified Gram negative bacterial species, *E. coli* (57.8%), was the predominant followed by *Klebsiella* spp. (31.4%). This concords with other studies [3, 4, 9, 12, 13]. *E. coli and K. pneumoniae* have emerged as serious pathogens worldwide. UTI occurs when *E. coli* and/or other gastrointestinal tract flora enters urinary tract [14].

Antibiotics play crucial role in treating such infections as long as the etiological bacteria is susceptible to the antibiotic activity. Thus, determining accurate antibiotic susceptibility is essential in the clinical care of bacterial infections. Bacteria capable of acquiring resistance demands more attention [15]. Most scholars throughout the world are reporting increasing AMR.

Most children are treated empirically before laboratory findings to avoid complications [9]. Both *E. coli* and *Klebsiella* spp. showed high resistance to ampicillin. This concord with other studies [3, 4, 9, 12, 13]. Susceptibility of *E. coli* and *K. pneumoniae* against different cephalosporins was similar. About 60% isolates were resistance to cephalosporins thus cephalosporins can be treatment option but cannot be relied as reserve drug or empiric drug. But higher generations of cephalosporins could promise effectiveness as shown by lower resistance against cefotaxime, about 40%. In contrast, *K. oxytoca* showed higher resistance to cephalosporins rendering ineffective for treatment. This further makes cephalosporins antibiotics unlikely for use as reserve 2nd line drug for treatment. Co-trimoxazole is preferred oral antibiotic for treatment of pediatric UTI but only half of the *E. coli* and *Klebsiella* spp. isolates were susceptible. Thus, sulfonamide antibiotics cannot be relied solely for treatment. Ofloxacin was ineffective against half of the *E. coli* isolates and one-fifth of *Klebsiella* spp. isolates. This makes fluoroquinolones antibiotics unreliable 2nd line choice as well. Meanwhile, nitrofurantoin and amikacin were effective against most of the *E. coli* and *Klebsiella* spp. isolates. Thus, nitrofurans antibiotics can be relied as 1st line choice for empiric treatment. Nitrofurans is also the economical option as it is comparatively cheaper. Alternatively, aminoglycosides antibiotics can also serve as reasonable choice. These drugs can thus be reserved for emergency use. Higher score of MDR (61.9%) among isolates underlines the higher risks associated with AMR and ask serious counter measures.

Inappropriate policies, poor surveillance, self-medication, poor diagnosis, poor quality of antibiotics, inadequate dose has all attributed to increase in AMR in recent years [16]. Inappropriate use of antibiotics in healthcare has increased the risk of AMR growth. This study was limited to phenotypic AMR detection excluding identification of different beta-lactamases producing bacteria. Genotyping characterization would provide further insight on AMR.

## Conclusions

Our findings outline high AMR among common uropathogens in tertiary setting. Varying epidemiology of AMR with time and locales has hindered the effective management of infections. Since, our study was confined to single healthcare setting, only the broader and effective surveillance can establish AMR data for effective clinical management of such pathogens. Such AMR data is not available at present in this region thus, our findings can be referenced for the treatment of UTIs in children.

## Abbreviations

AMR: Antimicrobial resistance
ATCC: American type culture collection
CFU: Colony forming unit
CLSI: Clinical Laboratory Standard Institute
MDR: Multidrug resistant
UTI: Urinary tract infection
